# Clinical Features of Infertile Men Carrying a Chromosome 9 Translocation

**DOI:** 10.1515/med-2019-0100

**Published:** 2019-11-10

**Authors:** Ruixue Wang, Yang Yu, Qiyuan Wang, Yuting Jiang, Linlin Li, Haibo Zhu, Ruizhi Liu, Hongguo Zhang

**Affiliations:** 1 Center for Reproductive Medicine and Center for Prenatal Diagnosis, First Hospital, Jilin University, 71 Xinmin Street, Chaoyang District, Changchun, Jilin Province 130021, China; 2 Experimental School of Changchun Jida Middle School, Changchun, China

**Keywords:** Male infertility, Chromosomal translocation, Chromosome 9, Breakpoint, Genetic counselling

## Abstract

Previous studies indicated that chromosome 9 translocations are involved in reduced male fertility and increased chance of miscarriage in the female partner. The aim of this study was to review the clinical features and genetic counselling requirements of infertile men carrying chromosome 9 translocations. This study analyzed fertile-age male carriers of chromosome 9 translocations, and included 12 clinical cases in our hospital. In our cases, three cases had oligozoospermia or severe oligozoospermia, while nine cases had normal semen. Of the latter nine cases, seven were associated with recurrent spontaneous abortions, and two produced a phenotypically normal child as confirmed by amniocentesis. Male chromosome 9 translocations and specific breakpoints from reported papers were searched using PubMed and CNKI database. A literature review identified 76 male patients who carried chromosome 9 translocations. Breakpoints at 9p12, 9p11, 9p10 and 9q34.1 were related to pregestational infertility, while breakpoints at 9p21, 9q10, 9q11, 9q13, 9q21.1, 9q22, 9q22.2, 9q22.3, 9q34, 9q34.2 and 9q34.3 exhibited gestational infertility. Chromosome translocations involving chromosome 9 lead to increased risk of miscarriage. Carriers of chromosome 9 translocations should be counselled to consider in vitro fertilization accompanied by preimplantation genetic diagnosis.

## 1 Introduction

Balanced reciprocal translocations are structural chromosomal abnormalities. Male carriers may have high rates of genetically unbalanced spermatozoa and exhibit impaired spermatogenesis, associated with frequent unbalanced embryos, male infertility or increased miscarriages [1, 2, 3]. However, clinical cases of normal male fertility with no history of related abortion can also be found for individuals with balanced translocations. Additionally, although in vitro fertilization accompanied by preimplantation genetic diagnosis (PGD) increased the chance of translocation carriers fathering a healthy child [4], some studies suggested that PGD did not make better for live birth rate and repeated miscarriage of couples with balanced translocations [5,6]. Natural conception is still a possible option for these carriers’ couples [7,8]. Hence, genetic counselling remains a challenge for carriers of balanced translocations.

Recently, we reported and reviewed the relationship between translocation breakpoints of chromosomes 2, 3, 5, and 6, and infertility for male carriers [9, 10, 11, 12, 13]. Previous studies indicated that chromosome 9 translocations are involved in reduced male fertility and increased chance of miscarriage in the female partner [4,14,15]. The chromosomes and specific breakpoints involved in the translocation are closely related to reproductive abnormalities [16,17]. Chromosomal translocation can increase the frequency of spermatozoa carrying an abnormal chromosome constitution, and some translocation breakpoints can disrupt important genes involved in spermatogenesis [10]. Testis-specific protein kinase 1 gene (*TESK1*) is located on chromosome 9p13.3 and is specifically expressed in testicular germ cells [18]. Thioredoxin domain-containing protein 8 gene (*TXNDC8*), mapped to chromosome 9q31.3, may be associated with late sperm maturation [19]. Additionally, chromosome 9 was the first chromosome found to be frequently associated with infertile patients [20]. Understanding the breakpoints on chromosome 9 with respect to providing genetic counselling for male infertility warrants further research.

The aim of this study is to identify potential correlations between clinical characteristics of male infertility and carriers of specific translocation breakpoints in chromosome 9.

## 2 Methods

Twelve male carriers of chromosome 9 translocations experiencing infertility or receiving counselling were recruited from the outpatient’s department at the Center for Reproductive Medicine, First Hospital of Jilin University, Changchun, China between July 2010 and December 2017. This study included all translocation cases involving chromosome 9, and excluded the patients with varicocele, ejaculatory duct obstruction and the other cause of infertility. Each patient underwent semen and cytogenetic analysis. Abortions due to the female factor were excluded. This study was approved by the Ethics Committee of the First Hospital of Jilin University, and written informed consent was provided by each patient.

For each patient, a semen sample obtained by masturbation after 3-7 days of abstinence was allowed to liquefy at room temperature, and was then analyzed using standard techniques recommended by the World Health Organization guidelines. Patients with oligozoospermia were diagnosed with a sperm count less than 15×106/ml in their last three semen samples (taken at intervals of 1–3 weeks). Oligozoospermia and severe oligozoospermia were defined as previously described [2]. Chromosome preparations were obtained from lymphocyte cultures derived from each patient. Karyotype analysis after G-banding of metaphase chromosomes followed our previously reported methods [11].

Male chromosome 9 translocations and specific breakpoints from reported papers were searched using PubMed, Google Scholar and CNKI database. The search keywords were “chromosome/ translocation/sperm” and “chromosome/translocation/ abortion”. This study included male cases of adult fertile-age, and excluded females and newborns carriers, those with complex chromosomal translocations, chimeras or bone marrow detection, and other cases without breakpoints involving chromosome 9 in the reported papers.

## 3 Results

**Figure 1:**
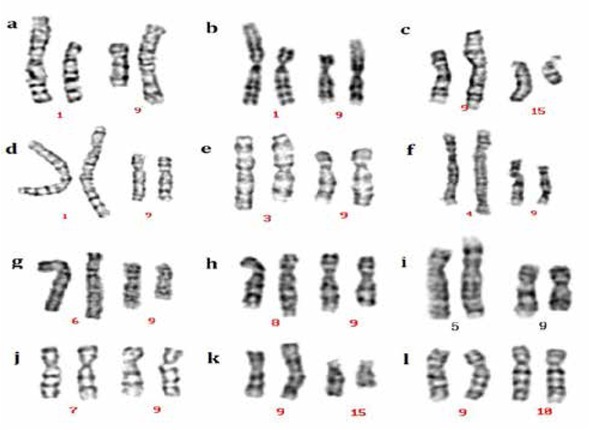
G-banding karyotypes of the 12 cases identified as possessing chromosome 9 translocations. a: t(1;9) (p22;p24); b: t(1;9) (p32;p24); c: t(9;15) (p13;q11); d: t(1;9); e: t(3;9); f: t(4;9); g: t(6;9); h: t(8;9); i: t(5;9); j: t(7;9); k: t(9;15) (p24;q22); l: t(9;10).

This study clinically examined a total of 12 men with chromosome 9 translocations. Karyotype results and G-banding karyotypes from these 12 patients are shown in Table 1 and Figure 1, respectively. Three cases had oligozoospermia or severe oligozoospermia (pregestational infertility), while nine cases had normal semen. Of the former three cases, the carrier with t(1; 9) (p32; p24) showed oligozoospermia, and the other two carriers manifested severe oligozoospermia. After genetic counselling and informed consent, the use of intracytoplasmic sperm injection combined with PGD should be carefully considered for these patients. Of the latter nine cases, it was evident that the carriers’ wife had a tendency to miscarry (gestational infertility); two cases with t(3;9)(q21;q22) and t(8;9)(q24;q32) produced a phenotypically normal child as confirmed by amniocentesis, respectively, and the other seven cases had experienced recurrent miscarriage. For these patients, PGD or prenatal diagnosis should be considered to improve pregnancy rates and reduce abortion rates.

**Table 1:** Karyotypes of chromosome 9 translocation carriers and their clinical features

Infertility type	Clinical findings	Karyotype	Figure No.
Pregestational	Oligozoospermia or severe oligozo- ospermia	46,XY,t(1;9)(p22;p24)	Figure 1a
		46,XY,t(1;9)(p32;p24)	Figure 1b
		46,XY,t(9;15)(p13;q11)	Figure 1c
Gestational	Normal sperm density; a history of miscarriage or Natural pregnancy	46,XY,t(1;9)(p36;q32)	Figure 1d
		46,XY,t(3;9)(q21;q22)	Figure 1e
		46,XY,t(4;9)(q35;p13)	Figure 1f
		46,XY,t(6;9)(q26;p13)	Figure 1g
		46,XY,t(8;9)(q24;q32)	Figure 1h
		46,XY,t(5;9)(p13;q22)	Figure 1i
		46,XY,t(7;9)(p10;q10)	Figure 1j
		46,XY,t(9;15)(p24;q22)	Figure 1k
		46,XY,t(9;10)(q21;q22)	Figure 1l

**Figure 2:**
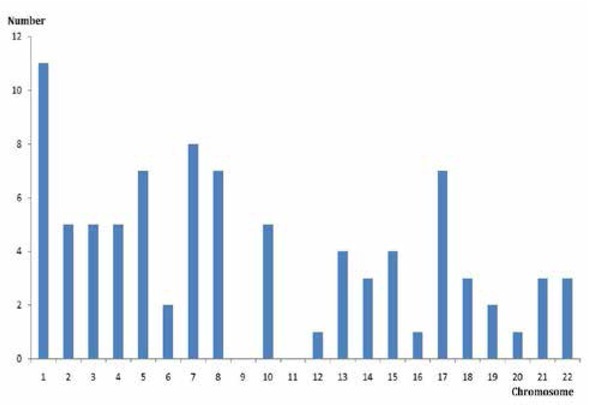
Distribution of chromosomes involved in translocations with chromosome 9.

From a review of the literature, clinical feature, karyotype, and specific breakpoints on chromosome 9 were collected and are summarized in Table 2. The reported paper included 76 carriers of chromosome 9 translocations. Combined with the 12 cases reported in this study, chromosome 1 (11 cases) is the most frequently involved with chromosome 9 translocation. In cases of male infertility, the distribution of other chromosomes involved in the translocation with chromosome 9 is shown in Figure 2. The distribution suggests that balanced translocation is closely related to male fertility, and multiple breakpoints on chromosome 1 are involved in these translocations. However, chromosome 11 was not found to be involved in translocation with chromosome 9.

**Table 2:** Breakpoints in chromosome 9 translocation carriers and clinical features reported in previous literature

Cases	Karyotype	Breakpoints	Clinical findings	Reference
1	t(1;9)	1q11;9p24	severe oligozoospermia	Yoshida et al., 1997 [75]
2	t(1;9)	1q43; 9p23	Oligoasthenozoospermia	Perrin et al., 2013 [45]
3	t(1;9)	1q11; 9p13	Azoospermia or severe oligozoospermia	Mierla et al., 2014 [14]
4	t(1;9)	1q12; 9q13	PGD	Zhang et al., 2014 [81]
5	t(1;9)	1q23; 9q22.3	Recurrent miscarriage	Dutta et al., 2011 [38]
6	t(1;9)	1q42.3; 9q22.3	Recurrent miscarriage	Sugiura-Ogasawara., 2008[56]
7	t(1;9)	1q22; 9q31	Infertility	Martin, 1992 [61]
8	t(1;9)	1p32.1; 9q34.3	Normal sperm count	Matsuda et al., 1992 [36]
9	t(2;9)	2q21; 9p22	Infertility	Martin et al., 1990 [60]
10	t(2;9)	2q32; 9q31	ICSI	Gekas et al., 2001 [52]
11	t(2;9)	2q33; 9q34	PGD	Findikli et al., 2003 [51]
12	t(2;9)	2q37; 9q22	2 fetal losses	Adamoli et al., 1986 [42]
13	t(2;9)	2q37.3; 9q12	Normal semen	Dul et al., 2012 [54]
14	t(3;9)	3p25; 9q32	Infertility	Honda et al., 1999 [64]
15	t(3;9)	3q21; 9q34	PGD	Findikli et al., 2003 [51]
16	t(3;9)	3q26.2; 9q32	Infertility	Honda et al., 1999 [64]
17	t(3;9)	3q28; 9q32	Azoospermia or severe oligozoospermia	Mierla et al., 2014 [14]
18	t(4;9)	4p15.2; 9p13	Recurrent spontaneous abortions	Celep et al., 2006 [78]
19	t(4;9)	4q23.2; 9q22.3	Recurrent pregnancy loss	Kochhar et al., 2013 [15]
20	t(4;9)	4q25; 9p22	Infertility	Moretti et al., 2009 [73]
21	t(4;9)	4q31.1; 9p24	Recurrent spontaneous abortions	Celep et al., 2006 [78]
22	t(5;9)	5p15.1; 9q22.1	Primary infertility	Vozdova et al., 2013 [4]
23	t(5;9)	5p13; 9q22	PGD	Zhang et al., 2014 [81]
24	t(5;9)	5p12; 9p11	Infertility	Pellestor et al., 2001 [66]
25	t(5;9)	5q10; 9q10	Infertility	Rouen et al., 2017 [35]
26	t(5;9)	5q23.2; 9q22.3	Spontaneous abortion	Stephenson et al., 2006 [48]
27	t(5;9)	5q23.3; 9p24	Repeated miscarriages	Iyer et al., 2007 [55]
28	t(6;9)	6p12; 9q13	PGD, No pregnancy	Escudero et al., 2003 [76]
29	t(7;9)	7p15.2; 9q34.1	Primary infertility	Vozdova et al., 2013 [4]
30	t(7;9)	7p14.2; 9q32	Recurrent miscarriage	Pundir et al., 2016 [57]
31	t(7;9)	7p13; 9p23	ICSI	Gekas et al., 2001 [52]
32	t(7;9)	7p13; 9q21	Teratozoospermia	Rouen et al., 2013[39]
33	t(7;9)	7q31; 9q34	Early miscarriage	Olszewska et al., 2017 [43]
34	t(7;9)	7q33; 9p21	Infertility	Pellestor et al., 1997 [63]
35	t(7;9)	7q36.2; 9p21.2	Normal semen	Wiland et al., 2008 [74]
36	t(8;9)	8p21; 9p24	Spontaneous abortions	Bourrouillou et al., 1986 [47]
37	t(8;9)	8p21; 9q34	Early miscarriage	Olszewska et al., 2017 [43]
38	t(8;9)	8q24.2; 9q32	Infertility	Estop et al., 1998 [68]
39	t(8;9)	8q24.3; 9p21.2	Severe oligozoospermia;	Olszewska et al., 2017 [43]
40	t(8;9)	8q24.2; 9q32	Infertility	Estop et al., 2000 [65]
41	t(8;9)	8q24.3; 9p24	Infertility	Ferfouri et al., 2013 [71]
42	t(9;10)	9q11; 10p11.1	Repetitive abortions	Rives et al., 2003 [67]
43	t(9;10)	9q12; 10q26	Azoospermia or severe oligozoospermia	Mierla et al., 2014 [14]
44	t(9;10)	9q34; 10q11	Infertility	Martin, 1988 [59]
45	t(9;10)	9q34.3; 10q24.1	PGD	Ko et al., 2010 [40]
46	t(9;12)	9p22; 12q22	Recurrent fetal wastage	Fryns et al., 1998 [79]
47	t(9;13)	9p23; 13q21.1	PGD	Ko et al., 2010 [40]
48	t(9;13)	9q21.1; 13q21.2	Infertility	Martin et al., 1995 [62]
49	t(9;13)	9q31; 13q34	Recurrent pregnancy loss	Kochhar et al., 2013 [15]
50	t(9;13)	9q32; 13q32	Recurrent miscarriage	Sugiura-Ogasawara., 2008 [56]
51	t(9;14)	9p13; 14q13	Normal semen	Wiland et al., 2008 [74]
52	t(9;14)	9q22.1; 14q12	Infertility	Gada Saxena et al., 2012 [80]
53	t(9;14)	9q32; 14p11.2	Severe oligozoospermia	Brugnon et al., 2006 [49]
54	t(9;15)	9p10; 15q10	Oligoasthenoteratozoospermia	Aydos et al., 2006 [72]
55	t(9;15)	9q32; 15q24	Multiple Abortions	Castle et al., 1988 [41]
56	t(9;16)	9q34.2; 16p12	Normal semen	Douet-Guilbert et al.,2005 [50]
57	t(9;17)	9p13; 17q21.3	Normal sperm count	Perrin et al., 2009 [77]
58	t(9;17)	9p13; 17q21.3	Infertility	Benet et al., 2005 [70]
59	t(9;17)	9p12; 17q24	Oligozoospermia	Antonelli et al., 2000 [46]
60	t(9;17)	9q12; 17p12	Infertility	Benet et al., 2005 [70]
61	t(9;17)	9q21; 17p11.2	PGD, Delivery	Kyu Lim et al., 2004 [37]
62	t(9;17)	9q22; 17p13	Normal semen	Brugnon et al., 2006 [49]
63	t(9;17)	9q22.1; 17p13.1	Repeated spontaneous abortions	Ghazaey et al., 2015 [3]
64	t(9;18)	9p12; 18q12.1	Infertility	Pellestor et al., 1989 [44]
65	t(9;18)	9q22.2; 18p11.31	Recurrent miscarriage	Pundir et al., 2016 [57]
66	t(9;18)	9q32; 18q23	Recurrent miscarriage	Pundir et al., 2016 [57]
67	t(9;19)	9q10; 19p10	Increased aneuploid sperm	Godo et al., 2013 [17]
68	t(9;19)	9q10; 19p10	Not recorded	Anton et al., 2008 [53]
69	t(9;20)	9p24; 20q13.1	Oligozoospermia and astenozoospermia	Anton et al., 2008 [53]
70	t(9;20)	9q13.4;20p11.2	Normal sperm count	Yoshida et al., 1997 [75]
71	t(9;21)	9p21; 21q22	Recurrent abortion	Gaboon et al., 2015 [58]
72	t(9;21)	9p13; 21q22.1	PGD	Zhang et al., 2014 [81]
73	t(9;21)	9q11; 21q10	IVF	Findikli et al., 2003 [51]
74	t(9;22)	9q21; 22q11.2	Oligospermia and asthenospermia	Perrin et al., 2009 [77]
75	t(9;22)	9q21; 22q11.2	Oligozoospermia	Douet-Guilbert et al.,2005 [50]
76	t(9;22)	9q21; 22q11.2	Infertility	Morel et al., 2004 [69]

The breakpoints at 9q32, 9p24 and 9p13 were observed in 12 cases (13.6%), 10 cases (11.4%) and 9 cases (10.2%) respectively. The breakpoints at 9p12, 9p11, 9p10 and 9q34.1 were related to pregestational infertility, while breakpoints at 9p21, 9q10, 9q11, 9q13, 9q21.1, 9q22, 9q22.2, 9q22.3, 9q34, 9q34.2 and 9q34.3 exhibited gestational infertility. Other breakpoints were found with cases of either pregestational or gestational infertility (Table 3).

**Table 3:** Incidence of breakpoints on chromosome 9

Breakpoints	Number of patients with pre-gestational infertility	Number of p atients with gestational infertility	Total (%)
p24	5	5	10(11.4%)
p23	2	1	3(3.4%)
p22	2	1	3(3.4%)
p21.2	1	1	2(2.3%)
p21		2	2(2.3%)
p13	2	7	9(10.2%)
p12	2		2(2.3%)
p11	1		1(1.1%)
p10	1		1(1.1%)
q10		3	3(3.4%)
q11		2	2(2.3%)
q12	1	2	3(3.4%)
q13		2	2(2.3%)
q13.4		1	1(1.1%)
q21	4	2	6(6.8%)
q21.1		1	1(1.1%)
q22		5	5(5.7%)
q22.1	1	2	3(3.4%)
q22.2		1	1(1.1%)
q22.3		4	4(4.5%)
q31	2	1	3(3.4%)
q32	3	9	12(13.6%)
q34		5	5 (5.7 %)
q34.1	1		1 (1.1 %)
q34.2		1	1 (1.1 %)
q34.3		2	2 (2.3%)

## 4 Discussion

This study reports the karyotype and clinical manifestations of 12 cases with chromosome 9 translocations. Three cases had oligozoospermia or severe oligozoospermia, seven cases were associated with recurrent spontaneous abortions, and two cases each produced a phenotypically normal child (confirmed by amniocentesis). Pregestational and gestational infertility are the most typical two types for infertile male [21]. This study included three cases that exhibited pregestational infertility, and seven cases that exhibited gestational infertility. These patients may consider intracytoplasmic sperm injection or PGD combined within vitro fertilization to reduce subsequent miscarriage rate. The two cases that produced a phenotypically normal child indicated that carriers have a chance of natural conception. The live birth rate in patients with chromosomal translocations choosing to conceive naturally was reported to be 37–63% for the first pregnancy, and then a cumulative rate of 65–83% [22].

To study the role of breakpoints on chromosome 9 in male infertility, the previously published literatures were reviewed. The clinical findings and karyotype regarding chromosome 9 are shown in Table 2. For all carriers from our study and reported literature, the most common chromosome and breakpoint involved chromosome 9 translocation were t(1;9) (12.5%) and 9q32 (13.6%) respectively. Chromosome 1 was most involved with chromosome 9 translocation in this study. For translocation carriers involved in chromosome 1, the clinical phenotype is more likely to be related to chromosome 1. Previous literatures have reported that there are more genes related to spermatogenesis on chromosome 1 [23,24]. The breakpoints on chromosome 1 could interfere with spermatogenesis, leading to azoospermia. In genetic counselling, the breakpoints on chromosome 1 should be considered for the translocation carriers involved in t (1; 9).

Breakpoints at 9p12, 9p11, 9p10 and 9q34.1 were found with pregestational infertility, while breakpoints at 9p21, 9q10, 9q11, 9q13, 9q21.1, 9q22, 9q22.2, 9q22.3, 9q34, 9q34.2 and 9q34.3 exhibited gestational infertility. Therefore, more chromosome 9 breakpoints exhibited gestational (compared with pregestational) infertility, in which the carriers’ wife had a tendency to miscarry. Other breakpoints were found with cases of pregestational or gestational infertility. Previous studies have shown that certain genes on chromosome 9 are involved in spermatogenesis. For example, the aquaporin 7 gene (*AQP7*), located on chromosome 9p13.3, is expressed during the late stages of spermatogenesis [25]. Spermatogenic failure 8 gene (*SPGF8*), mapped to chromosome 9q33.3, is associated with severe spermatogenic failure [26]. The outer dense fiber of sperm tails 2 gene (*ODF2*), mapped to chromosome 9q34.11, has a key role in the formation of sperm flagella [27]. Doublesex-and mab3-related transcription factor 1 (*DMRT1*) and *DMRT3*, mapped to chromosome 9p24.3, are expressed in germ cell and Sertoli cells [28]. Relaxin 1 (*RLN 1*) and RLN2, mapped to chromosome 9p24.1, are highly expressed in the prostate and testis [29]. Lysine-specific demethylase 4c (*KDM4C*) located on chromosome 9p24.1 regulates histone modifications and androgen receptor function [30]. Testis expressed 48 (TEX 48) and TEX53, mapped to chromosome 9q32, are testis-specific genes [31]. The specific function of these genes needs further investigation. Kim et al [32] reported that breakpoints at 9p22; 9p11.2, 9q21.2 and 9q22 were found with cases of impaired spermatogenesis, and breakpoints at 9p23.24, 9p23, 9p12 and 9q22 were associated with recurrent abortion. The key difference between the above paper and our current study is that their subjects had complex chromosomal rearrangements. The carrier is more likely to have normal sperm in semen when they involve balanced translocation of two chromosomes. For the carriers of complex chromosomal rearrangements, the probability of abnormal spermatogenesis increase greatly.

Male carriers of chromosome translocations are phenotypically normal, but may produce genetically unbalanced spermatozoa, leading to unbalanced embryos and miscarriage. Translocations may alter the process of spermatogenesis, resulting in azoospermia or oligospermia [33]. To explain the associated clinical pregestational or gestational infertility, three hypotheses have been proposed, including a break within a gene, a positional effect, and cryptic deletion or duplication [34]. During genetic counselling, physicians should consider the breakpoints involved in the translocation. When receiving genetic counselling, the carriers of chromosome 9 translocations should consider suitable reproductive options, including continued attempts at natural conception or in vitro fertilization accompanied by PGD.

Limitations of this study include the small number of carriers of chromosome 9 translocations, and the lack of detailed research regarding the specific molecular effects of each translocation by molecular-cytogenetic methods. According to our knowledge, this study is the first review of male carriers involved in chromosome 9 translocation published in previous literature, which will provide reference for clinical genetic counselling.

## 5 Conclusion

In the present study, the most common breakpoints involving chromosome 9 translocation were t(1;9) and 9q32 respectively. Most breakpoints at chromosome 9 exhibited gestational infertility. Carriers of chromosome 9 translocations should be counselled to consider in vitro fertilization accompanied by PGD.
